# Experiences and perceptions of online continuing professional development among clinicians in sub-Saharan Africa

**DOI:** 10.1186/s12960-017-0266-4

**Published:** 2017-12-29

**Authors:** Caryl Feldacker, Sheena Jacob, Michael H. Chung, Anya Nartker, H. Nina Kim

**Affiliations:** 1 International Training and Education Center for Health (I-TECH), 325 Ninth Avenue, Box 359932, Seattle, WA United States of America; 2 Department of Global Health, University of Washington, 325 Ninth Avenue, Box 357965, Seattle, WA United States of America; 3 Department of Medicine, University of Washington, 325 Ninth Avenue, Box 359930, Seattle, WA United States of America; 4 Department of Epidemiology, University of Washington, 325 Ninth Avenue, Box 359909, Seattle, WA United States of America

**Keywords:** Medical education, e-Learning, Online education, Healthcare worker quality, Sub-Saharan Africa, Continuing professional development

## Abstract

**Background:**

Limitations in healthcare worker (HCW) capacity compound the burden of dual TB and HIV epidemics in sub-Saharan Africa. To fill gaps in knowledge and skills, effective continuing profession development (CPD) initiatives are needed to support practicing HCWs reach high standards of care. e-learning opportunities can bring expert knowledge to HCWs in the field and provide a flexible learning option adaptable to local settings. Few studies provide insight into HCW experiences with online CPD in the developing country context.

**Methods:**

An online survey using both close-ended and free response was conducted to HCWs in sub-Saharan Africa who completed the University of Washington (UW) School of Medicine online graduate course, “Clinical Management of HIV.” Associations between respondent characteristics (age, gender, rural/urban, job title) and learning preferences, course barriers, and facilitators with an emphasis on online courses were examined using chi-square. Covariates significant at the *p* < 0.05 were analyzed using multivariable logistic regression. Responses to open-ended comments were analyzed using simplified grounded theory.

**Results:**

Of 2,299 former students, 464 (20%) HCWs completed surveys from 13 countries: about half were women. Physicians (33%), nurses (27%), and clinical officers (30%) responded mostly from urban areas (67%) and public institutions (69%). Sixty-two percent accessed the online course from work, noting that slow (55%) or limited (41%) internet as well as lack of time (53%) were barriers to course completion. Women (*p* < 0.001) and HCWs under age 40 (*p* = 0.007) were more likely to prefer learning through mentorship than men or older HCWs. Respondents favored group discussion (46%), case studies (42%), and self-paced Internet/computer-based learning (39%) and clinical mentorship (37%) when asked to choose 3 preferred learning modalities. Free-response comments offered additional positive insights into the appeal of online courses by noting the knowledge gains, the flexibility of format, a desire for recognition of course completion, and a request for additional online coursework.

**Conclusions:**

Online CPD opportunities were accepted across a diverse group of HCWs from sub-Saharan Africa and should be expanded to provide more flexible opportunities for self-initiated learning; however, these need to be responsive to the limited resources of those who seek these courses.

**Electronic supplementary material:**

The online version of this article (10.1186/s12960-017-0266-4) contains supplementary material, which is available to authorized users.

## Background

In many parts of sub-Saharan Africa, where the burden of TB and HIV epidemics are the highest, healthcare systems are severely challenged by a critical shortage of healthcare workers (HCWs) [1, 2]. Diverse efforts are urgently needed not only to increase the number of trained HCWs but also to expand and strengthen workforce capacity such that existing HCWs are “fit for purpose and fit for practice” [3]. Continuing Professional Development (CPD) refers to the purposeful and ideally ongoing education that HCWs undertake after completion of basic training to maintain their core competencies and update their knowledge, skills, and practices [4]. CPD activities can range from traditional classroom learning to independent online coursework with the same goal of creating a qualified workforce capable of successfully meeting the needs of the populations they serve.

In the field of HIV and TB, the pace of change in treatment options and diagnosis make the flexibility and efficiency of online education attractive. e-Learning utilizes technology to bridge the distance gap between qualified technical experts and the HCWs who could benefit from additional clinical training. e-Learning can take various forms, ranging from web-based or online courses, digital libraries, offline modules on diverse electronic devices, or live video teleconferencing [5–7]. Particularly in resource-constrained settings where expert faculty are scarce or inaccessible, e-learning has emerged as a powerful tool to enable fewer faculty to reach a wider audience of learners and to permit students to remain in their worksites, learn at their own pace, and reduce travel costs associated with more traditional training courses [6, 8]. e-Learning may also help reduce training-related absenteeism, improve HCW retention, and reduce the impact of training on family life [9].

Internet-based learning for health professionals has been shown, in systematic reviews of studies from mostly resource-rich settings, to have a positive impact on skills, knowledge, behavior, and patient care compared with no intervention and at least comparable effectiveness to traditional non-internet-based instruction [10–12]. Less is known about the impact or acceptability of online CPD in resource-limited settings. One comprehensive literature review of online learning in low- or middle-income countries suggested enthusiasm and favorable outcomes for e-learning [6]; however, the review focused predominantly on pre-service training and noted that most published educational efforts were aimed at physicians (58%) versus other cadres [6]. Physicians however comprise a small minority of HCWs in many African countries, with a density of fewer than 5 per 10 000 persons [13]. Nurses and midwives constitute more than half of the national health workforce in these settings and can exceed the number of physicians by 3–5-fold [13]. Task-shifting, the delegation of medical duties from higher to lower cadres or new cadres, has emerged as a major coping strategy to address this disproportionate shortage of physicians during the scale-up of antiretroviral therapy in sub-Saharan Africa [14]. Whether online CPD has acceptance across the diversity of health worker cadres in such regions remains unclear [8, 15].

Moreover, existing literature on online learning, and online CPD in particular, has historically emphasized program implementation or learning outcomes over learner preferences or acceptance [16]. Regardless of the type of learning activity, an assessment of learning needs and evaluation of learner experience are critical not only for those who deliver CPD but for the clinicians themselves, as such reflection can enhance the recognition of gaps in knowledge or skills and promote changes in practice [17]. The experience and learning preferences of diverse HCWs from resource-limited settings with online CPD is not well documented [8]. We, therefore, conducted an online survey of HCWs from 13 different countries in sub-Saharan Africa who completed the University of Washington (UW) online graduate course, “Clinical Management of HIV” [18]. We ascertained learning preferences as well as identified key barriers and facilitators to learning, with an emphasis on their online CPD experience.

## Methods

We conducted an online, semi-quantitative, structured survey from September to October 2016 using a convenience sample of HCWs who enrolled as students in the UW Department of Global Health e-learning course, “Clinical Management for HIV” [18], an online course offered annually to HCWs in 20–30 countries in Asia, Africa, South America, and the Caribbean. Invitations to the online survey were emailed to all students in sub-Saharan Africa who participated in the course from 2012 to 2016 and identified as medical or nursing professionals. In brief, this e-learning course is led by UW faculty and offered to both HCWs in low- and middle-income countries as well as to UW graduate students. Ten 2-h sessions cover a variety HIV clinical topics. HCWs view recorded lectures asynchronously in a group and are asked to complete weekly homework assignments (online case studies) and quizzes. A certificate is given to for students who attend ≥ 80% of lectures, complete ≥ 90% of homework, and achieve a cumulative ≥ 70% score on quizzes.

### Data collection, management, and analysis

The survey was developed in English, pre-tested and modified based on pilot testing with 30 respondents. Our questions were adapted from an example needs assessment survey provided by the African Health Professions Regulatory Collaborative as part of a “Toolkit for Developing a National CPD Framework” in April 2014 [19]. Although the survey was not formally validated, it was designed by experts in the CPD field as part of a package of tools to help practitioners and policy makers identify more effective CPD teaching methods and prioritize CPD learning needs of nurses and midwives [20]. Our final survey consisted of 14 questions with an estimated completion time of 10 min and included multiple-choice questions that asked about the participant’s demographics, clinical setting, professional background, course experience, and learning preferences and challenges with taking the course. See Additional file 1 for our online survey tool. We ended the survey with an open-ended question inviting them to share further thoughts or experiences related to CPD. The survey was administered and delivered via an online tool Survey Monkey (www.surveymonkey.com/). Respondent data was subsequently imported and summarized using Stata 14 (College Station, TX) [21]. We examined associations between respondent characteristics (age, years of experience, job title, country) and preferences on learning modalities and between setting (rural or urban) and answers regarding barriers to online CPD using chi-squared testing. We analyzed covariates that were significant at the *p* < 0.05 level using multivariable logistic regression [22]. The responses to the open-ended request for comments were coded in Microsoft Excel using a simplified grounded theory approach [22, 23], an iterative approach to generate themes from the qualitative data. First, one qualitative researcher created a set of overarching codes from the answer categories illustrated from the data. Then, upon a second review by the same researcher, supplemental themes were added to both complement and provide contradictory insight. Responses were categorized and summary tallies shared with other members of the research team to help ensure neutrality and representativeness of the findings.

## Results

### Survey respondents

Among 2696 students from sub-Saharan African countries who participated in the UW e-learning course, “Clinical Management of HIV,” from 2012 to 2016, we sent survey invitations via email to the 2299 (85%) former students who identified as medical or nursing professionals. Of those invited, 464 (20%) HCWs completed surveys. Participating countries included Kenya (43%), Namibia (24%), Nigeria (14%), Zambia (7%), Cameroon (3%), Ethiopia (2%), and Botswana (1%). The respondents were evenly split by gender. The majority (70%) were between 20 and 40 years of age (Table 1). The respondents included physicians (33%), nurses (27%), and clinical officers (30%). This distribution of countries, health professions, gender, and age was comparable to the distribution in the course overall from 2012 to 2016 and during years when gender/age data was collected. Most worked in urban areas (67%) and public institutions (69%); 62% worked in hospital-based settings. Sixty-two percent had accessed the online course from work, 24% from home, and 7% from an Internet café.

**Table 1:** Characteristics of the online survey respondents

Participants	Total (*N* = 458)
Gender	
Male	228 (50%)
Female	230 (50%)
Age in years	
20–30	114 (25%)
31–40	208 (45%)
41–50	90 (20%)
> 50	46 (10%)
Years’ experience as HCW	
1–5	111 (24%)
6–10	157 (34%)
11–15	78 (17%)
16–20	45 (10%)
21–25	30 (7%)
> 25	37 (8%)
Job title	
Physician	153 (33%)
Registered nurse	124 (27%)
Clinical/medical officer	137 (30%)
Nurse assistant	2 (< 1%)
Other	42 (9%)
Location	
Urban	309 (67%)
Rural	151 (33%)
Institution	
Public	321 (69%)
Private	70 (15%)
Faith-based	35 (8%)
Other	38 (8%)
Facility	
National/central hospital	85 (19%)
Regional hospital	71 (15%)
District hospital	130 (28%)
Urban health center	45 (10%)
Rural health center	50 (11%)
Other	79 (17%)

*HCW* health care worker

### Learning preferences

**Fig. 1:**
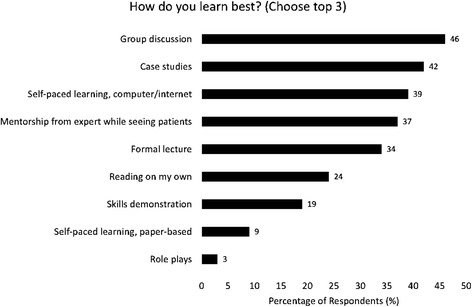
Preferred learning modalities among online survey respondents challenges noted by healthcare workers in

Among responses to the question, “What is your preferred location for CPD access?” just over half (52%) reported online, with another 31% the workplace and 17% outside of work. The respondents favored a range of learning modalities in response to the question, “How do you best learn? (Choose top 3)” (Fig. 1). Group discussion was selected most often (46%), followed closely by case studies (42%) and self-paced Internet/computer-based learning (39%). Many others selected mentorship (37%) and more formal lecture (34%).

### Motivators and barriers to access

**Fig. 2:**
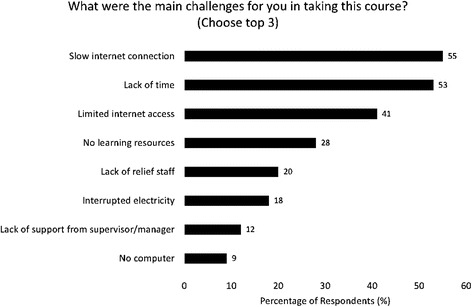
Main challenges to CPD access among online survey participants

The vast majority (73%) responded that they took the course because it met their learning interests, with small proportions citing convenience (11%), affiliation with an academic institution (7%), certificate (5%), and requirement (4%) as their primary reason for participation. Several barriers to CPD were noted in the responses to the question, “What were the main challenges with taking this online course (Choose up to 3 top choices)?” A majority of the respondents cited slow or limited Internet access (55 and 41%, respectively) and lack of time (53%) as their main challenges to accessing the course (see Fig. 2).

### Association between HCW background and responses

The selection of online as the preferred site of CPD activity did not differ by gender, age, job title, or experience and ranged from 47 to 55% across groups defined by these characteristics. Women were more likely than men to favor mentorship as a learning modality (46 versus 28%, *p* < 0.001). This association between gender and mentorship was not explained by job title, which had no association with learning modality. HCWs younger than 40 years were also more likely to favor mentorship than HCWs older than 40 years (41 versus 28%, *p* = 0.007). In a multivariable model that included age (dichotomized as age less than or equal to 40 years versus age greater than 40 years) and gender, these were both found to be independently associated with mentorship; adjusted odds ratio (aOR) for older age was 0.50 (95% confidence interval [CI] 0.31 to 0.78, *p* = 0.002) and aOR for men was 0.41 (95% CI 0.31 to 0.78, *p* < 0.001). We found no association between age, gender, or job title with other learning modalities. We found no statistically significant difference in responses regarding barriers among rural versus urban HCWs nor differences in either learning preferences or challenges based on country.

### Additional comments

In a final open-ended query, we invited the 464 respondents to provide any additional comments on their online CPD experience, and 207 (45%) individuals responded. We summarize these comments by key themes in Table 2. Of the 207 comments, 64% were positive regarding their online course experience. Many specified their knowledge gains from the course content (16%) and wanted expanded course options (13%), including other HIV- and TB-related content.

**Table 2:** Distribution of comment categories

Comment category	Total (*n*)	Percent
General positive comment (thank you, great course, etc.)	133	64.3
Knowledge gained through course	32	15.5
More courses desired	27	13.0
Desire for certificate	21	10.1
Difficult access to course	15	7.2
Generic suggestions for course improvement	14	6.8
Want free/low-cost courses	13	6.3
Time for CPD course was too short	10	4.8
Improved care/service quality	10	4.8
Want option to participate individually	9	4.3
Convenience of online	3	1.4
Mentors are needed	3	1.4

Some noted the benefit of the course for flexibility, appreciating that the course was available when the health professionals could make time in their schedules.

“CPD is a way of improving your knowledge outside of the classroom and the online delivery makes it convenient for busy professionals like myself because I can always catch up in my spare time after working hours. Thank you for the opportunity.”

“It was a rewarding experience and for busy professionals like me it offers flexibility and can fit into your schedule.”

“An opportunity to enhance learning while working and earning a living.”

Others noted that they felt the course provided knowledge that could improve the quality of their service delivery.

“The training is doing a good job in improving patient safety and quality care delivery. This is a plus for the advancement of healthcare services in the developing countries.”

“It is a great way to develop and or improve skills and knowledge in other fields and to ensure one is updated in global best practices. That has been my experience.”

“I gained valuable knowledge through continuing professional development which has helped me in providing quality health care.”

The appeal of online learning for resource-constrained settings was mentioned by the respondents.

“The CPD is very vital for health workers in sub-Saharan Africa. Please roll it out to many people as possible as many people are unable to go for further studies due to limited resources.”

“It is an excellent method of learning for expanding knowledge base/capacity building. Please continue providing us this type of learning especially to some of us who are in resource limited settings.”

The interactive component with online commentary and exchange was also seen as beneficial as participants enjoyed learning from their peers in other locations and countries.

“It was a great experience interacting with people from different countries. It introduced me to online learning and I can't just get enough of it. Everyday am learning something new from online education.”

However, for those in the developing country context to fully take advantage of online courses, access must improve, especially in rural areas. The barriers of access and slow Internet connectivity appeared repeatedly.

“The idea is very good, but it can only work in the urban areas where there is light and internet services.”

“I would prefer to take the course in person. Because there is poor internet access especially in my area.”

It was also suggested that such courses should come with the recognition for successful efforts in the form of certificates of completion (10%) that would be recognized by local organizations. Still, on top of the certificates, several wanted more advanced offerings.

“It would be great if we could have diploma courses and not only certificates. Most participants after completing the certificate wanted to learn more.”

Others noted (6%) that the courses should have lower cost or be free and that an expanded time frame (5%) to complete the course content would help with both time and technology constraints.

## Discussion

From a large cross-section of HCWs in sub-Saharan Africa who accessed an advanced online HIV course, more than half of the respondents preferred to access CPD online (52%) to other CPD locations (e.g., work) and this preference was not associated with HCW characteristics (gender, age, job title). This was so even though many cited limitations with Internet access (slow (55%) or limited (41%) Internet) as major barriers to CPD access. A majority of HCWs identified lack of time as a major barrier (53%), a factor that would challenge CPD uptake in any format. Women and HCWs under age 40 were more likely to prefer learning through clinical mentorship than men or older HCWs. Free-response comments offered additional positive insights into the appeal of online courses by noting the knowledge gains, the flexibility of format, a desire for recognition of course completion, and a request for additional online coursework. Few studies exist that evaluate online CPD for developing country HCWs, especially in the critical context of HIV/TB. Access to continuing education has been shown to promote retention of HCWs at rural facilities in sub-Saharan Africa [24]. Our findings provide others interested in expanding CPD options a better understanding of the learning preferences of those already showing interest in CPD—an audience more likely to adopt additional CPD courses that meet their needs.

We found that in addition to self-paced learning via computer/Internet, many of our respondents favored clinical mentorship, case-based teaching, and group discussion, emphasizing the importance of interactive modalities of learning. This feedback suggests that distance education could benefit from collaborative technologies such as chat boxes or discussion boards. Studies have shown higher levels of learner satisfaction and improvements in knowledge, self-awareness, and changes in practice with these participatory aspects of e-learning in medicine and other disciplines [25, 26]. Moreover, other studies suggest that, even in an era of increasing uptake of e-learning [27], in-person learning activities with peers and experts remain popular among practicing clinicians and, at times, may be favored over technology-based learning [28–31]. This underscores the importance of providing multifaceted, or blended, options for the diversity of learning needs and preferences [32].

Our findings also suggest CPD that takes advantage of multiple modalities, i.e., blended learning opportunities, are worthy of consideration. Blended learning combines a variety of learning methods (e.g., in-person, online, print, and social media) and learning environments (e.g., instructor-led, teamwork, peer-to-peer interaction, self-study, and individual work) [33] but has not been as extensively studied as other modalities of learning [12]. The UW HIV course encouraged a site facilitator and peer group discussion model for the lecture viewing blended with online learning. This model gives HCWs access to high-quality learning materials and expert faculty and allows content to be localized [18, 34]. An offline e-learning component, complemented by a mix of mentorship and on-site or mobile group discussions, could offer a convenient, self-paced, and flexible modality for learners that can be adapted for the local context at the work site.

Moreover, our course was offered at the workplace where Internet access may be faster or more reliable. It is critical to recognize that the vast majority of households still have limited access to computers and Internet in sub-Saharan Africa [15, 35]. For those who want individual or more flexible learning options, CPD conducted offline on mobile devices, such as tablets or phones [36] or via CD-ROM [37], offers the potential for providing self-paced e-learning opportunities without requiring consistent access to the Internet. This may in part address the slow or poor access to Internet expressed by many respondents.

Our study has several limitations. First, we employed a convenience sample of predominantly urban HCWs who previously accessed online learning and still had Internet access. This group may have better computer literacy and opportunities than other HCWs who live in more rural settings [8]. Indeed, the two larger source countries for our participants, Kenya and Nigeria, lead among African countries with the greatest Internet usage [38]. Our response rate was low but comparable to that reported in similar survey-based studies of health professionals [39, 40]. This however may have led to selection bias; those who did not respond may have had different online or learning challenges not considered here. Lastly, the survey employed for this exercise was not formally validated; however, we believe that the questions utilized were informed by experts in the CPD field and, therefore, are useful to help inform the future direction of online CPD.

## Conclusion

Our findings are illustrative of preferences and considerations related to online CPD among a large cohort of medical and nursing professionals in sub-Saharan Africa, informing strategies for improved CPD options for HCWs in these settings. Online CPD opportunities were widely accepted across a diverse group of HCWs from multiple countries and recognized as a flexible opportunity for self-initiated learning. Limited HCW time and Internet access remain the main barriers to online CPD participation; these systemic challenges require sustained efforts and continued innovation to overcome. Future studies should examine the unique CPD needs and challenges of rural HCWs who may have less access to consistent clinical oversight and other CPD support, including similar online opportunities. Developing practical, e-learning solutions for more easily accessible, quality CPD programming for all HCWs should remain a priority for those involved in the continued improvement of the healthcare workforce in sub-Saharan Africa.

## Additional file

Additional file 1: Online CPD survey. (DOCX 16 kb)

## Abbreviations

CPD: Continuing Professional Development
HCW: Healthcare worker
UW: University of Washington
