# Supplementary material for: Experiences and perceptions of online continuing professional development among clinicians in sub-Saharan Africa
Source: Hum Resour Health. 2017 Dec 29;15:89. doi: 10.1186/s12960-017-0266-4 (PMC5747038; doi:10.1186/s12960-017-0266-4)
Supplement: Additional file 1: — Online CPD survey. (DOCX 16 kb) [file 12960_2017_266_MOESM1_ESM.docx]

Additional file 1: Online CPD Survey

Continuing Professional Development (CPD) Needs Assessment
*Online Survey: University of Washington HIV Clinical Management Course Users*

Given your prior experience with the University of Washington HIV Clinical Management course, you are invited to take part in a research survey about continuing professional development (CPD) and online distance learning for health care workers in sub-Saharan Africa. We want to gain a better understanding of this area to help support HIV- and TB-related service delivery in Africa. This survey should take no more than 10 minutes.

Taking part in this study is completely voluntary. Choosing not to participate will not adversely affect your relationship with anyone at the University of Washington or your institution. Your responses will be received anonymously, kept strictly confidential and stored in secure computer files. Any report of this research that is made public will not include your name or any other individual information by which you could be identified. If you have questions, you can contact the lead researcher, Dr. Nina Kim, at hyangkim@uw.edu.

Clicking the “Next” button below indicates that you are 18 years of age or older, and indicates your consent to participate in this survey.

Section A: Sociodemographic Data

1. In what type of institution do you work?
2. Public
3. Private
4. Faith-based
5. Other
6. Is your clinic in a rural or urban area?
7. Urban
8. Rural
9. What type of health facility do you work in?
10. National/central hospital
11. Regional hospital
12. District hospital
13. Urban health center
14. Rural health center
15. Other
16. What is your gender?
17. Male
18. Female
19. What is your age?
20. 20–30 years
21. 31–40 years
22. 41–50 years
23. Greater than 50 years
24. How many years of health-related work experience do you have?
25. 1–5 years
26. 6–10 years
27. 11–15 years
28. 16–20 years
29. 21–25 years
30. Greater than 25 years
31. Which option best describes your current job at your workplace?
32. Physician
33. Clinical officer/medical assistant
34. Registered nurse midwife/registered nurse
35. Enrolled nurse/enrolled nurse midwife
36. Nurse assistant
37. Other

Section B: Continuing Professional Development (CPD) and Distance Learning

The following set of questions refer to your experience and preferences in this area.

1. Where did you most commonly access the course?
2. Home
3. Workplace
4. Internet café
5. University library
6. Other
7. Why did you decide to take the course?
8. Convenience of online learning
9. Certificate at end of course
10. Course addressed my learning needs
11. Its affiliation with an academic institution of learning
12. How best do you learn?
13. Formal lectures
14. Group discussions
15. Case studies
16. Skills demonstrations
17. Self-paced learning modules, paper-based
18. Self-paced learning modules, computer-/Internet-based
19. Reading journal articles
20. Role plays
21. Being mentored by an expert in a clinic setting while seeing patients
22. What were the main challenges with taking this online course? (Choose up to 3 top choices)
23. Lack of time
24. Interrupted electricity
25. Limited access to Internet connection
26. Slow Internet connection
27. No computer/laptop
28. Lack of relief staff
29. Limited learning resources (e.g., library, up-to-date textbooks/journals)
30. Lack of support from supervisor/manager
31. Where should CPD activities ideally be done?
32. My workplace
33. Other facility (not workplace)
34. Online
35. If you have another chance to take an HIV- or TB-related CPD course in the future, which delivery would you prefer?
36. Online
37. In person
38. Is there anything else you would like to tell us about your CPD experience(s)?
39. No
40. Yes. Please specify the challenges below: ____________________________________________________________________________________________________________________________________________________________________________________________________________________________________________________________________________________________________________
